# Biomechanical investigation of long spinal fusion models using three-dimensional finite element analysis

**DOI:** 10.1186/s12891-023-06290-4

**Published:** 2023-03-08

**Authors:** Norihiro Oku, Satoru Demura, Daisuke Tawara, Satoshi Kato, Kazuya Shinmura, Noriaki Yokogawa, Noritaka Yonezawa, Takaki Shimizu, Ryo Kitagawa, Makoto Handa, Annen Ryohei, Hiroyuki Tsuchiya

**Affiliations:** 1 Department of Orthopaedic Surgery, Graduate School of Medical Sciences, Kanazawa University, 13-1, Takara-Machi, Kanazawa, Ishikawa 920-8641 Japan; 2 Department of Mechanical and Systems Engineering, Faculty of Science and Technology, Ryukoku University, Shiga, Japan

**Keywords:** Finite element analysis, Long spinal fusion, Spinal sagittal balance, Von Mises stress, Upper instrumented vertebra, Pedicle screw, Transverse hook, Adult spinal deformity, Osteoporosis, Sagittal vertical axis

## Abstract

**Background:**

This study represents the first finite element (FE) analysis of long-instrumented spinal fusion from the thoracic vertebrae to the pelvis in the context of adult spinal deformity (ASD) with osteoporosis. We aimed to evaluate the von Mises stress in long spinal instrumentation for models that differ in terms of spinal balance, fusion length, and implant type.

**Methods:**

In this three-dimensional FE analysis, FE models were developed based on computed tomography images from a patient with osteoporosis. The von Mises stress was compared for three different sagittal vertical axes (SVAs) (0, 50, and 100 mm), two different fusion lengths (from the pelvis to the second [T2-S2AI] or 10th thoracic vertebra [T10-S2AI]), and two different types of implants (pedicle screw or transverse hook) in the upper instrumented vertebra (UIV). We created 12 models based on combinations of these conditions.

**Results:**

The overall von Mises stress was 3.1 times higher on the vertebrae and 3.9 times higher on implants for the 50-mm SVA models than that for the 0-mm SVA models. Similarly, the values were 5.0 times higher on the vertebrae and 6.9 times higher on implants for the 100-mm SVA models than that for the 0-mm SVA models. Higher SVA was associated with greater stress below the fourth lumbar vertebrae and implants. In the T2-S2AI models, the peaks of vertebral stress were observed at the UIV, at the apex of kyphosis, and below the lower lumbar spine. In the T10-S2AI models, the peaks of stress were observed at the UIV and below the lower lumbar region. The von Mises stress in the UIV was also higher for the screw models than for the hook models.

**Conclusion:**

Higher SVA is associated with greater von Mises stress on the vertebrae and implants. The stress on the UIV is greater for the T10-S2AI models than for the T2-S2AI models. Using transverse hooks instead of screws at the UIV may reduce stress in patients with osteoporosis.

## Background

The number of patients with adult spinal deformities (ASDs), such as degenerative scoliosis and kyphosis, continues to increase [1]. Severe spinal deformities tend to cause pain due to the deformity itself and can profoundly restrict activities of daily living.

ASDs are commonly treated via corrective spinal fusion using different types of metal implants, which can be made of various materials. Nonetheless, many limitations of ASD surgery remain to be resolved. For example, in most patients with ASDs, spinal malalignment often calls for long spinal fusion surgery from the thoracic spine to the pelvis. However, extensive fusion has been associated with various complications, such as loosening or dislocation of implants and adjacent vertebral fractures around the implants, especially in patients with osteoporosis. Therefore, minimizing these complications is mandatory. At present, there is no clear consensus on the optimal spinal alignment, fusion length, and implant types for reducing the risk of implant-related failure. In addition, few studies have investigated biomechanical stress in long spinal fusion models using three-dimensional finite element analysis (3D-FEA) [2].

In this study, we utilized 3D-FEA to investigate the changes in von Mises stress on the vertebrae and implants among osteoporotic long-instrumented spinal fusion models that differed with respect to spinal alignment, fusion length, and implant type.

## Materials and methods

### Creation of a whole-spine model extending from the thoracic spine to the pelvis

In this study, we used 3D-FEA software (Mechanical Finder [MF], version 10.0, Extended Edition, RCCM Co. Ltd., Tokyo, Japan). We analyzed computed tomography (CT) data obtained from a 64-year-old woman with a bone mineral density of 0.717 g/cm^2^. CT was performed at 0.625-mm intervals from the cervical spine to the pelvis, and the CT data were transferred to MF. The ethics committee of our institute approved the use of this patient’s CT data (Approval No. 1748). We created 3D-FEA bone models from the first thoracic vertebra (T1) to the pelvis by extracting bone contour lines using MF. The models consisted of tetrahedral elements with a length of 1.4 mm.

We derived the mass density of the bone ρ (g/cm^3^) from the CT value (Hounsfield Unit, HU) and calculated the non-homogeneous Young's modulus distribution based on Keyak's formula [3, 4] to determine the material properties of the finite elements. The Young's modulus E (MPa) is expressed as indicated in Formula 1, as follows:

1 $$E= \left\{\begin{array}{c} 0.001 \left(\rho =0\right) \\ 33900{\rho }^{2.20} \left(0<\rho \le 0.27\right) \\ 5307\rho +469 \left(0.27<\rho <0.6\right)\\ 10200{\rho }^{2.01} \left(0.6\le \rho \right) \end{array}\right.$$

**Fig. 1:**
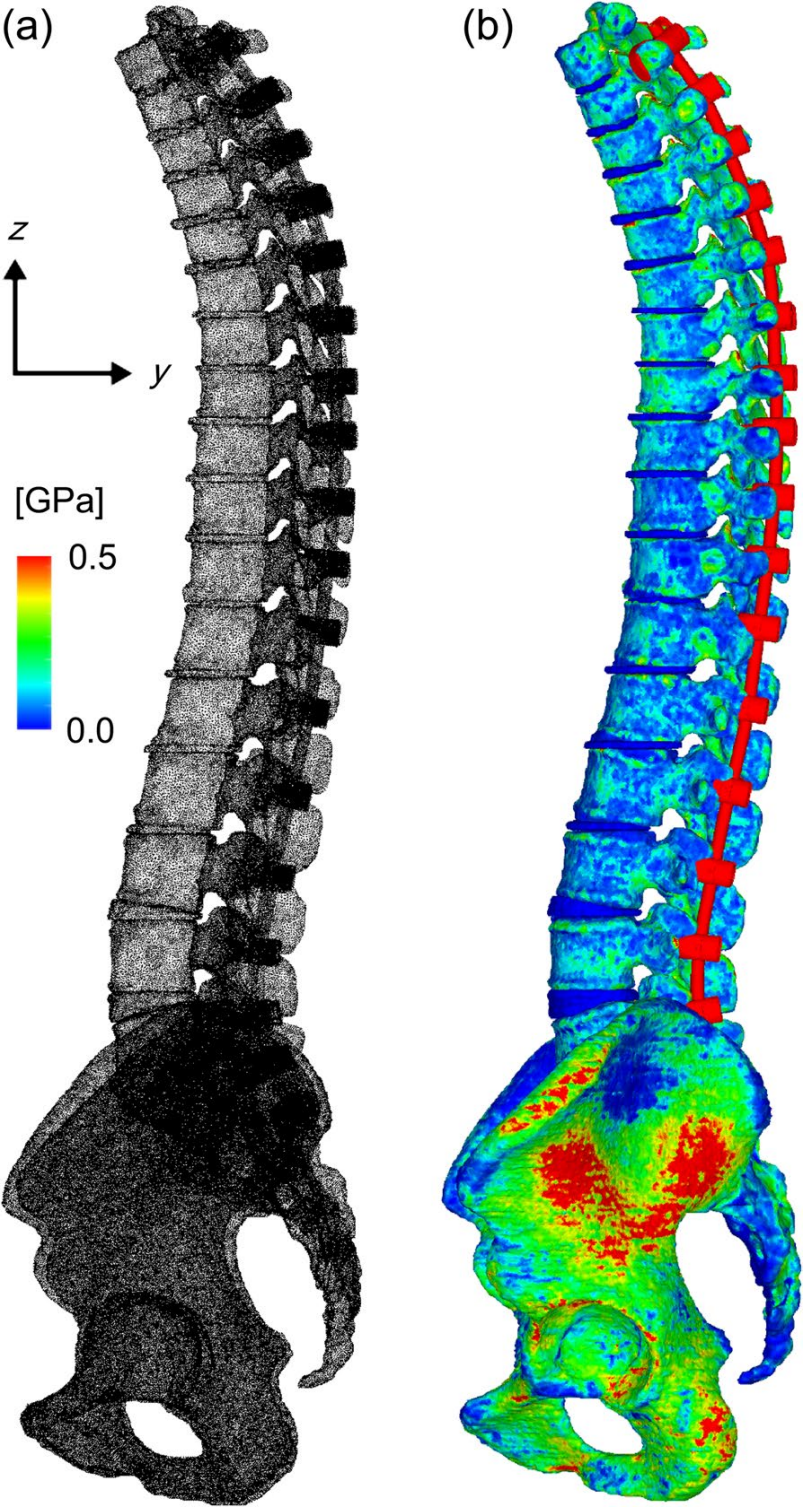
Element segmentation diagram of multi-vertebrae and Young’s modulus distribution diagram. **A** FE models of spinal fusion. **B** Heterogeneous distribution of Young's modulus, *E*. FE, finite element; *E*, Young’s modulus (MPa)

Figures 1A and B show the diagrams of element decomposition and non-homogeneous Young's modulus distribution, respectively. Table 1 shows the Young's modulus and Poisson's ratio values for the vertebral body and intervertebral discs.

**Table 1:** Young's modulus and Poisson's ratio of each element

	Young’s modulus	Poisson’s ratio	Element type
Cortical bone	Determined by Formula 1	0.4	Tetrahedral
Cancellous bone	Determined by Formula 1	0.4	Tetrahedral
Disc	7.5 MPa	0.4	Tetrahedral

We obtained imaging data for the implants used in the actual surgery via micro-CT, which included a pedicle screw (PS; screw), an S2-alar-iliac screw (S2AI), and a transverse hook (TH; hook). Computer-aided design (CAD) data for these implants were created based on the CT data. The CAD software SOLIDWORKS^®^ (Concord, MA, USA) was used to create the implant models. The diameter of the screw analyzed in the current study was 5.5 mm, with a length ranging from 35 to 40 mm. The diameter and length of the S2AI screw were 8.5 and 90 mm, respectively. The diameter of the rod was 6.0 mm. After creating the implant models, we imported the bone and implant models into MF and created the FE models of long spinal fusion by combining these data. All screws and rods were fixed. The contact condition between implant and bone was set as bonding contact.

### Creation of instrumented spinal fusion models and load constraint conditions

#### Spinal balance

**Fig. 2:**
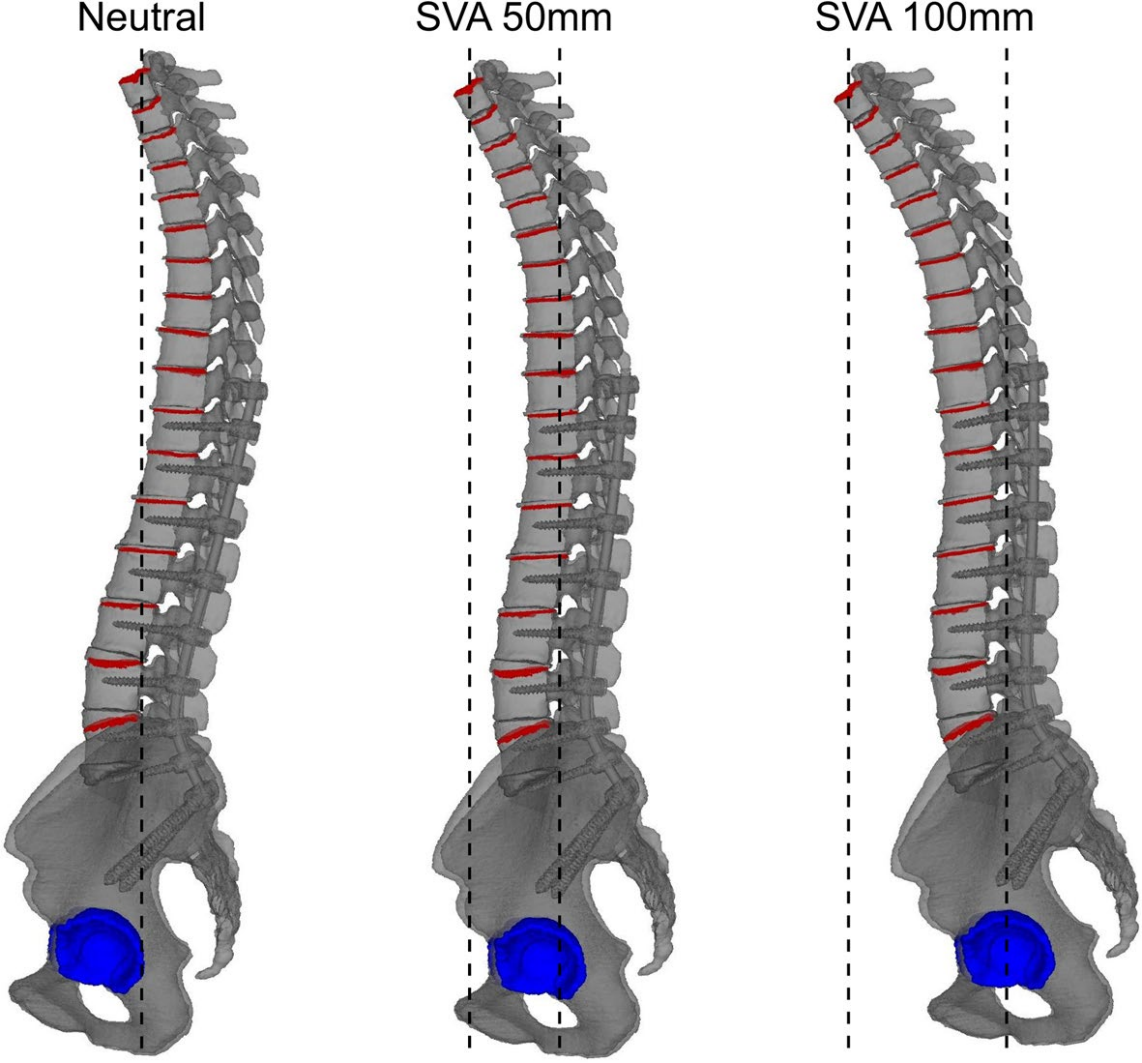
Examples of different models of spinal alignment. SVA, sagittal vertical axis

We evaluated the following three different patterns of standing sagittal vertical axes (SVAs): neutral [SVA 0 mm], SVA 50 mm, and SVA 100 mm (Fig. 2). We constrained fully acetabular regions of spinal fusion model, and created several models by forward tilting the spine centered on the constrained acetabular so that the SVA was 0 mm, 50 mm, and 100 mm.

#### Fusion length

**Fig. 3:**
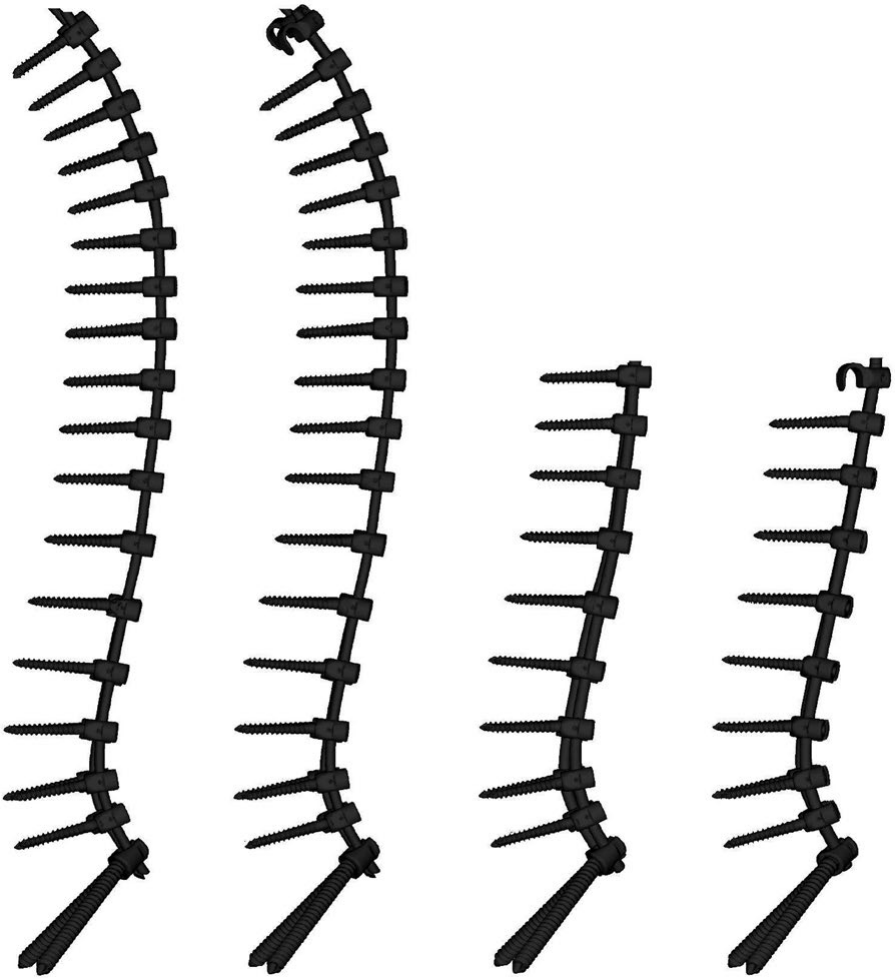
Examples of models with different fusion lengths and implants at the UIV. UIV, upper instrumented vertebra

We analyzed fusion length using the following two different conditions: from the second thoracic vertebra to the pelvis (T2-S2AI) and from the 10th thoracic vertebra to the pelvis (T10-S2AI) (Fig. 3).

#### Implant types in upper instrumented vertebrae (UIV)

We created the models by applying screw in each vertebra. We also developed the models using a hook at the UIV, instead of a screw (Fig. 3). Accordingly, we evaluated 12 models based on combinations of these conditions. Table 2 shows the number of nodes and tetrahedral elements for each model.

**Table 2:** Number of elements and nodes in each model

Fusion length	Implant types in UIV	Spinal balance	Number of nodes	Number of elements
T2-S2AI	Pedicle screw (All PS)	Neutral (SVA 0 mm)	1,544,795	7,481,374
		SVA 50 mm		
		SVA 100 mm		
	Transverse hook (TH)	Neutral (SVA 0 mm)	1,591,719	7,747,207
		SVA 50 mm		
		SVA 100 mm		
T10-S2AI	Pedicle screw (All PS)	Neutral (SVA 0 mm)	1,381,316	6,662,229
		SVA 50 mm		
		SVA 100 mm		
	Transverse hook (TH)	Neutral (SVA 0 mm)	1,377,463	6,628,824
		SVA 50 mm		
		SVA 100 mm		

### Boundary and loading conditions

For the boundary conditions, acetabular regions were constrained, a distributed load of 1,200 N was applied according to the number of nodes in the upper endplates of each vertebral body from T1 to L5, and static elastic stress analysis was performed. The loading method was applied based on that described in a previous study [5].

### Data extraction

For all 12 models, we selected and extracted the meshes of each vertebra from T2 to the sacrum. The total stress of the vertebral mesh was added and equalized according to the volume of each vertebra. We collected data on stress from the screws at each vertebra. The rod was extracted separately, and the von Mises stress was evaluated. We analyzed the von Mises stress on each vertebra and implant, focusing on the differences in spinal balance, fusion length, and implants in UIV. Previous studies have validated the use of FEM analysis for examining von Mises stress in the lumbar vertebrae [6–8].

## Results

### Comparison of von Mises stress in different spinal balance conditions

**Fig. 4:**
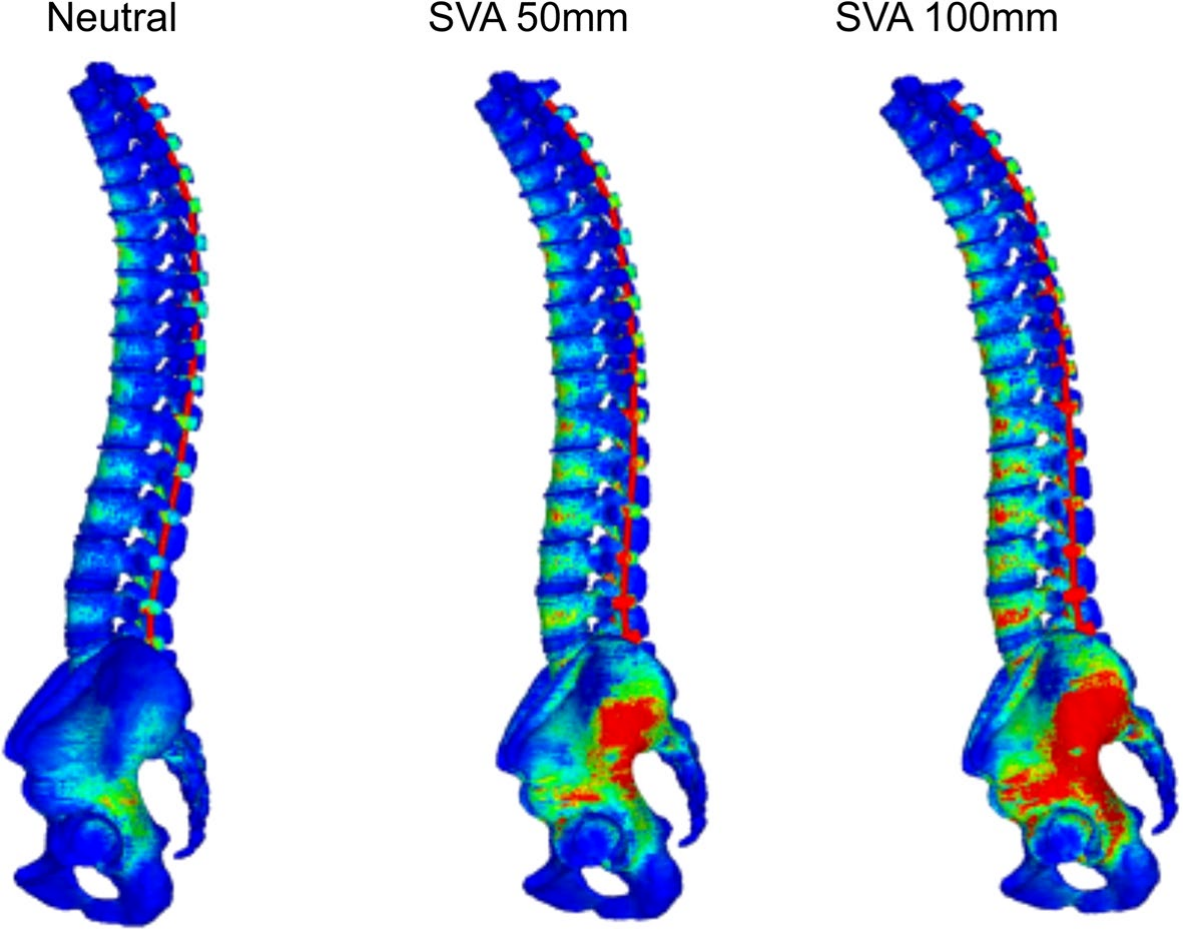
Examples of a contour diagram (T2-S2AI with all PS). SVA, sagittal vertical axis; T2-S2AI, from the second thoracic vertebra to the pelvis; PS, pedicle screw

Figure 4 shows a contour diagram demonstrating the von Mises stress values. A larger SVA was associated with greater stress on the vertebrae and implants. The von Mises stress was 2.6 to 3.1 times higher on the vertebrae and 3.7 to 3.9 times higher on the implants for the 50-mm SVA models than for the 0-mm SVA models. Similarly, the values were 4.3 to 5.0 times higher on the vertebrae and 6.3 to 6.9 times higher on the implants for the 100-mm SVA models than for the 0-mm SVA models.

### Comparison of von Mises stress among the different fusion lengths

**Fig. 5:**
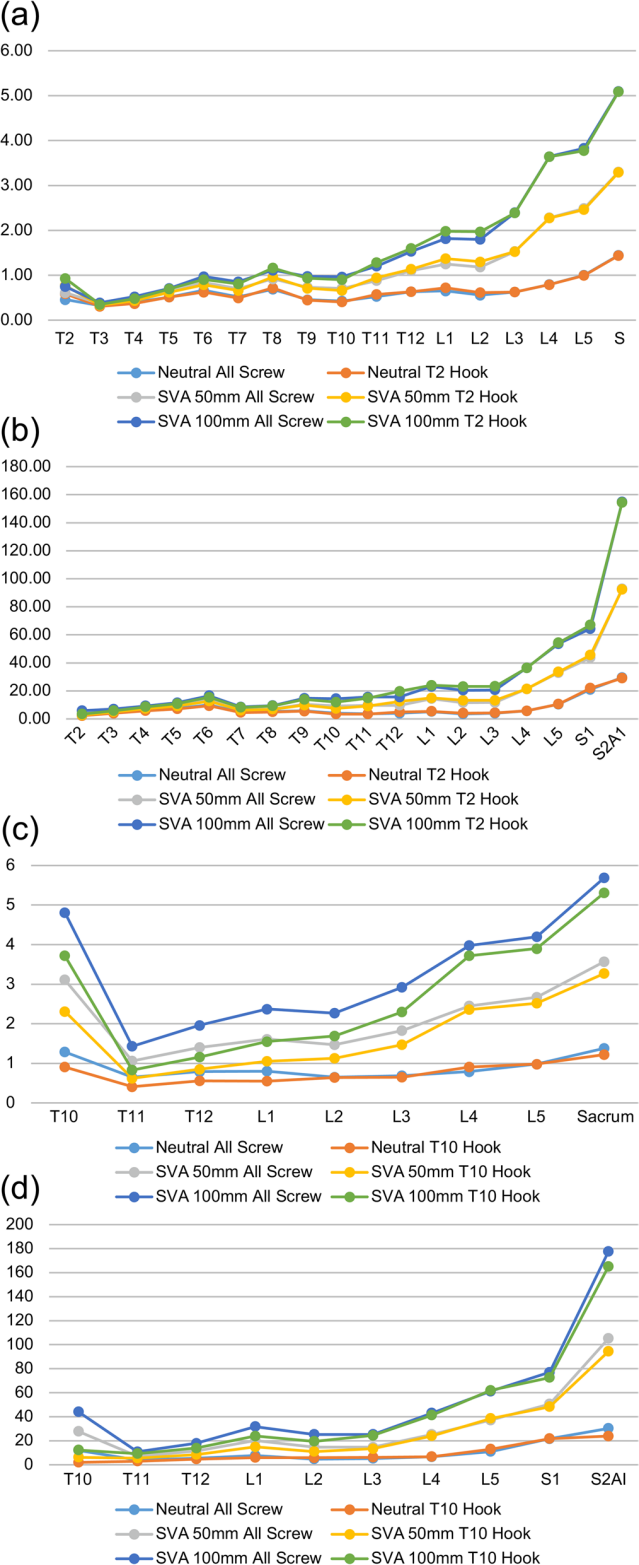
Stress distribution of the vertebrae and implants. **A** Stress distribution of the vertebrae in the T2-S2AI models. **B** Stress distribution of the implants in the T2-S2A models. **C** Stress distribution of the vertebrae in the T10-S2AI models. **D** Stress distribution of the implants in the T10-S2AI models. SVA, sagittal vertical axis; T2-S2AI, from the second thoracic vertebra to the pelvis; T10-S2AI, from the 10th thoracic vertebra to the pelvis

Figure 5 shows the von Mises stress distribution of each model. The T2-S2AI models had three peaks of vertebral stress, which are as follows: the first peak was at T2 (UIV), the second was at the apex of kyphosis, and the third was below the lower lumbar spine (Fig. 5A). The T2-S2AI models had two peaks of stress in implants, which are as follows: the first peak was near the apex of kyphosis, whereas the second was near the lower lumbar region (Fig. 5B). In all 100-mm SVA-screw models, the von Mises stress values in the vertebra at T2 and L5 were 0.7 and 3.8 MPa, respectively, and those of the implants at T2 and L5 were 5.9 and 53.6 MPa, respectively. Thus, the von Mises stress notably increased below the fourth lumbar vertebra. The T10-S2AI models had two peaks of stress in the vertebrae and implants. The first peak was at the UIV, and the second was below the lower lumbar region (Fig. 5C, D). The von Mises stress of the T10-S2AI models increased in the lower lumbar spine, as observed for the T2-S2AI models.

In all 100-mm SVA-screw models, the von Mises stress on the vertebrae at T10, T11, and L5 were 4.8, 1.4, and 4.2 MPa, respectively, and those on the implants at T10, T11, and L5 were 44.0, 10.8, and 61.1 MPa, respectively.

### Comparison of von Mises stress among the different types of implants at UIV

In the T10-S2AI models, the von Mises stress on both the vertebrae and implants at UIV was higher for the screw models than for the hook models. The von Mises stress on the vertebrae and implants was 1.4 and 5.9 times greater, respectively. In the T2-S2AI models, the von Mises stress on the vertebrae did not differ between the screw and hook models.

## Discussion

In this study, we employed 3D-FEA to examine the von Mises stress on the vertebrae and implants among different osteoporotic long-instrumented spinal fusion models. Our results demonstrated that higher SVA was associated with greater stress in all spinal models. The von Mises stress on the vertebrae and implants in the lower lumbar spine was also seven times greater for the 100-mm SVA models than for the 0-mm SVA models. Another study also reported that implant stress increased by 20%–42% in the gravity loading tests among patients with mechanical failures, when compared to that observed in patients without failure [9]. The substantially poorer SVA results in our study may explain the relatively greater stress relative to that observed in previous studies. Thus, preoperative planning to attain an optimal SVA is crucial for reducing postoperative complications due to von Mises stress in patients scheduled for spinal fusion surgery.

In the current study, von Mises stress distributions differed depending on the fusion length, and the T2-S2AI models exhibited lesser stress than the T10-S2AI models. There are two possible explanations for this result. First, a long fusion length may have dispersed and reduced the von Mises stress in each vertebra and implant. Second, spinal weight above UIV was lower for the T2-S2AI models than for the T10-S2AI models, and von Mises stress at UIV under gravity may have consequently been lower for the T2-S2AI models. Choosing the upper thoracic vertebrae as the site of the UIV has been reported to decrease the risk of proximal junctional problems [10]. We hypothesized that longer fusion length and more instrumented segments would also contribute to reducing proximal junctional problems.

The von Mises stress at UIV and adjacent vertebrae was higher in the T10-S2AI models than in the T2-S2AI models. The von Mises stress around UIV also increased more in the screw condition than in the hook condition. Hence, our results indicate that the risk of proximal junctional problems increases if the UIV is in the thoracolumbar transition area or when a screw is applied in the UIV. A previous study demonstrated that using a hook at the UIV instead of a screw decreased the biomechanical indices thought to be involved in the pathomechanisms of proximal junctional kyphosis [11]. Another study also reported that using a hook in the UIV may prevent proximal junctional problems more effectively than using a screw in clinical settings [12]. We hypothesized that using a hook on the cortical bone would be more effective in preventing proximal junctional problems than using a screw in the cancellous bone, given that the cancellous bone is much weaker than the cortical bone in patients with osteoporosis, meaning that it is less able to tolerate von Mises stress.

This study had some limitations. First, the load constraint conditions do not precisely match the conditions in the actual living bodies. There has been no consensus on the appropriate load constraint conditions to be used in finite element method (FEM) models. Second, the present model did not include soft tissue elements, such as ligaments and muscles. We believe that specific software capable of dealing with soft tissues, such as the AnyBody system (AnyBody Technology, Aalborg, Denmark), would solve this problem and allow for the development of FEM models that include soft tissues. Finally, our study evaluated bone models created using data from a single patient, highlighting the need for further studies to analyze various bone qualities.

## Conclusion

The current findings indicate that larger SVA results in greater von Mises stress on the vertebrae and implants. The von Mises stress on the UIV was also greater for the T10-S2AI models than for the T2-S2AI models. Using a hook instead of a screw at the UIV may help to reduce stress more effectively.

## Data Availability

The datasets used and/or analysed during the current study are available from the corresponding author on reasonable request.

## Abbreviations

ASD: Adult spinal deformity
3D: FEA, three-dimensional finite element analysis
FE: Finite element
FEM: Finite element method
MF: Mechanical Finder
CT: Computed tomography
T1: First thoracic vertebra
HU: Hounsfield Unit
PS: Pedicle screw
S2AI: S2-alar-iliac screw
TH: Transverse hook
CAD: Computer-aided design
SVA: Sagittal vertical axis
T2-S2AI: From the second thoracic vertebra to the pelvis
T10-S2AI: From the 10th thoracic vertebra to the pelvis
UIV: Upper instrumented vertebrae
